# *In vivo* tracking transplanted cardiomyocytes derived from human induced pluripotent stem cells using nuclear medicine imaging

**DOI:** 10.3389/fcvm.2023.1261330

**Published:** 2023-09-07

**Authors:** Yukihiro Saito, Naoko Nose, Toshihiro Iida, Kaoru Akazawa, Takayuki Kanno, Yuki Fujimoto, Takanori Sasaki, Masaru Akehi, Takahiro Higuchi, Satoshi Akagi, Masashi Yoshida, Toru Miyoshi, Hiroshi Ito, Kazufumi Nakamura

**Affiliations:** ^1^ Department of Cardiovascular Medicine, Okayama University Hospital, Okayama, Japan; ^2^ Molecular Imaging Project of RECTOR Program, Faculty of Medicine, Dentistry and Pharmaceutical Sciences, Okayama University, Okayama, Japan; ^3^ Department of Cardiovascular Medicine, Graduate School of Medicine, Dentistry and Pharmaceutical Sciences, Okayama University, Okayama, Japan; ^4^ Okayama Medical Innovation Center, Faculty of Medicine, Dentistry and Pharmaceutical Sciences, Okayama University, Okayama, Japan; ^5^ Department of Nuclear Medicine, University Hospital Würzburg, Würzburg, Germany; ^6^ Comprehensive Heart Failure Center, University Hospital Würzburg, Würzburg, Germany; ^7^ Department of Cardiovascular Medicine, Faculty of Medicine, Dentistry and Pharmaceutical Sciences, Okayama University, Okayama, Japan; ^8^ Department of Chronic Kidney Disease and Cardiovascular Disease, Faculty of Medicine, Dentistry and Pharmaceutical Sciences, Okayama University, Okayama, Japan; ^9^ Department of General Internal Medicine 3, Kawasaki Medical School, Okayama, Japan

**Keywords:** sodium/iodide symporter, human induced pluripotent stem cell-derived cardiomyocytes, single photon emission computed tomography, cell-based therapy, *in vivo* imaging

## Abstract

**Introduction:**

Transplantation of human induced pluripotent stem cell-derived cardiomyocytes (iPSC-CMs) is a promising treatment for heart failure. Information on long-term cell engraftment after transplantation is clinically important. However, clinically applicable evaluation methods have not yet been established.

**Methods:**

In this study, to noninvasively assess transplanted cell engraftment, human *SLC5A5*, which encodes a sodium/iodide symporter (NIS) that transports radioactive tracers such as ^125^I, ^18^F-tetrafluoroborate (TFB), and ^99m^Tc-pertechnetate (^99m^TcO_4_^−^), was transduced into human induced pluripotent stem cells (iPSCs), and nuclear medicine imaging was used to track engrafted human iPSC-CMs.

**Results:**

To evaluate the pluripotency of NIS-expressing human iPSCs, they were subcutaneously transplanted into immunodeficient rats. Teratomas were detected by ^99m^TcO_4_^−^ single photon emission computed tomography (SPECT/CT) imaging. NIS expression and the uptake ability of ^125^I were maintained in purified human iPSC-CMs. NIS-expressing human iPSC-CMs transplanted into immunodeficient rats could be detected over time using ^99m^TcO_4_^−^ SPECT/CT imaging. Unexpectedly, NIS expression affected cell proliferation of human iPSCs and iPSC-derived cells.

**Discussion:**

Such functionally designed iPSC-CMs have potential clinical applications as a noninvasive method of grafted cell evaluation, but further studies are needed to determine the effects of NIS transduction on cellular characteristics and functions.

## 1. Introduction

The number of patients with heart failure has become a global problem as the population ages, and the number of patients with atherosclerotic disease has increased due to saturated diets (1). Heart failure occurs when the heart cannot supply the blood necessary for physical activity due to myocardial cell loss and fibrosis. Advances in drug and device therapies for heart diseases have improved the prognosis of patients with heart failure (2). However, the patient number is still increasing. One reason might be the lack of a fundamental solution for cardiomyocyte loss.

In recent years, transplantation of cardiomyocytes derived from human induced pluripotent stem cells (iPSCs) has shown promise in replenishing lost cardiomyocytes and, a few clinical trials are ongoing (3, 4). However, no clinical method has been established to confirm the engraftment and viability of transplanted human iPSC-derived cardiomyocytes (iPSC-CMs). The inability to assess transplanted cardiomyocyte viability means that information is lacking to determine treatment efficacy, the need for re-transplantation, and the need for continued immunosuppressive agents, and establishing a non-invasive method to assess cell viability *in vivo* is a critical challenge to the success of cardiac regenerative therapy.

Magnetic resonance imaging (MRI) using superparamagnetic iron oxide (SPIO) and bioluminescence imaging (BLI) using the luciferin-luciferase reaction have been experimentally employed to evaluate the engraftment of transplanted cells. When SPIO-incorporated cells are transplanted and survival is evaluated by MRI, it is difficult to evaluate long-term cell viability because SPIO accumulated in macrophages that have phagocytosed the transplanted dead cells is detected by MRI (5, 6). BLI is often used in cell transplantation experiments with small animals. However, the problem of detection depth poses a barrier to its application in large animals, including human (7–9). In addition, since luciferase is a non-human-derived protein, the administration of luciferase-expressing cells can induce an immune response. In contrast, nuclear medicine imaging offers superior detection depth and spatial resolution compared with BLI (10). Templin et al. transduced a sodium/iodide symporter (NIS), an ion pump highly expressed in the thyroid and stomach that actively transports iodide into human iPSCs (11, 12). First, NIS-expressing human iPSCs were injected into infarcted piglet hearts treated with immunosuppressants. Next, ^123^I, a radioactive tracer that is taken up intracellularly via NIS, was injected into the coronary arteries, and the transplanted NIS-expressing human iPSC-derived endothelial cells were detected using single photon emission computed tomography/computed tomography (SPECT/CT). Ostrominski et al. also transduced NIS into rhesus iPSCs and transplanted cardiomyocytes derived from the NIS-expressing rhesus iPSCs into immunodeficient mice (13). Subsequently, ^18^F-tetrafluoroborate (TFB), a radioactive tracer taken up intracellularly via NIS (14), was administered intravenously, and the transplanted NIS-expressing rhesus iPSC-CMs were successfully detected using positron emission tomography/computed tomography (PET/CT). NIS is expressed in the thyroid and stomach and is not immunogenic. In addition, nuclear medicine imaging is already used in clinical practice, and the hurdles to its clinical application are likely to be lower than for other methods. However, there are no reports of studies evaluating the engraftment of human iPSC-CMs using nuclear medicine imaging, and further investigation using human iPSC-CMs is needed for clinical application.

In this study, we investigated whether transplanted NIS-expressing human iPSC-CMs could be tracked over time using ^99m^Tc-pertechnetate (^99m^TcO_4_^−^) SPECT/CT, which is more accessible than PET/CT. In addition, the effects of NIS transduction on cellular characteristics were investigated.

## 2. Materials and methods

### 2.1. Human induced pluripotent stem cell culture

A human iPSC line previously established from peripheral mononuclear cells obtained from a healthy subject employing episomal vectors (pCXLE-hOCT3/4-shp53-F, Cat.#. 27077; pCXLE-hSK, Cat.#. 27078; pCXLE-hUL, Cat.#. 27080; and pCXWB-EBNA1, Cat.#. 37624, Addgene, Watertown, MA, United states) was used in this study. Human iPSCs were maintained on iMatrix-511 (Cat.#. 892021, Matrixome, Osaka, Japan)-coated 6-well plates (Cat.#. 3516, Corning, Glendale, Arizona, United States) in StemFit AK02N (Cat.#. AK02N, Ajinomoto, Tokyo, Japan), and were passaged every 7 days.

### 2.2. Transduction of *SLC5A5* gene into human induced pluripotent stem cells

“pAAVS1_SA_loxP_PGK_*Neo*_loxP/CAG_IRES_*EGFP/AmpR*” that we previously generated was used for a donor vector (15). Human *SLC5A5* open reading frame (ORF) subcloned into pCMV6-XL5 vector (Cat.#. OHu19270C) was purchased from GenScript (Piscataway, NJ, United States). *SLC5A5* ORF was amplified by polymerase chain reaction (PCR), purified, and ligated with an XhoI-digested control donor vector using In-Fusion HD Cloning Kit w/Competent Cells (Cat.#. 639642, Clontech, Mountain View, CA, United States) to obtain “pAAVS1_SA_loxP_PGK_*Neo*_loxP/CAG_ *SLC5A5*_IRES_*EGFP/AmpR*”. The PCR primers are listed in Table 1.

**Table 1:** PCR primers.

Name		Sequence	Product size (bp)	Annealing temperature (°C)
Subcloning PCR				
Human *SLC5A5*	Forward	5′-TCATTTTGGCAAAGAATTCCACCGCCATGGAGGCCGTG-3′	1978	65
	Reverse	5′-ACGTAGCGGCCGCGATATCCTCAGAGGTTTGTCTCCTGCTGGTC-3′		
Flanking PCR				
Primer 1		5′-TGGGCTTTGCCACCCTATGCTGACA-3′		64
Primer 2		5′-ACAAGCAGAAGAACGGCATCAAGGTGAA-3′		
Primer 3		5′-ACCAACCATCCCTGTTTTCCTAGGACTGA-3′		

Human iPSCs were harvested using StemPro Accutase Cell Dissociation Reagent (Cat.#. A1110501, Thermo Fisher Scientific, Waltham, MA, United States). Ten micrograms of a non-linearized donor vector, 5 μg of AAVS1 1l TALEN (Cat.#. 35431, Addgene) and 5 μg of AAVS1 1R TALEN (Cat.#. 35432, Addgene) were transduced using Nucleofector Ⅱ/Program B-016 (Cat.#. AAD-1001N, Amaxa Biosystems, Nordrhein-Westfalen, Germany) and Amaxa Human Stem Cell Nucleofector Kit 1 (Cat.#. VAPH-5012, Lonza, Basel, Switzerland). The cells were treated with 50 μg/ml G-418 (Cat.#. 4727878001, Roche Applied Science, Penzberg. Germany) for 10 days. EGFP-positive colonies were picked up and expanded on iMatrix-511-coated plates using StemFit AK02N. The established clones were genotyped by PCR. The PCR primers used in this study are listed in Table 1.

### 2.3. *In vitro* radioactive tracer uptake study

^18^F-TFB was synthesized in-house as previously described (16). The radiochemical purity of ^18^F-TFB was consistently > 98%, with activity > 1.8 GBq/ml. ^125^I was supplied as Na^125^I (Cat.#. NEZ033l; PerkinElmer, Inc., Boston, MA, United States). The radiochemical purity of the radiolabeled compounds was greater than 99%.

^18^F-TFB and ^125^I uptake study using human iPSCs were performed as previously described (17) with minor modifications. In brief, human iPSCs were seeded on iMatrix-511-coated 24-well plates at 1 × 10^5^ cells/well and maintained in StemFit AK02N for 3 days. The cells were then washed twice with Hanks' balanced salt solution (HBSS, Cat.#. 14175079, Thermo Fisher Scientific), and incubated in 450 µl of HBSS with or without the NIS inhibitor NaClO_4_ (200 µM). 2.0 MBq/ml ^18^F-TFB and 0.8 MBq/ml ^125^I in 50 µl of HBSS were added to each well (*n* = 3), followed by incubation for 60 min (37°C, 5% CO_2_). The incubation buffer was removed, and ^18^F-TFB and ^125^I uptake into the cells was immediately terminated by adding 1 ml of ice-cold HBSS.

Human iPSC-CMs were seeded on iMatrix-511-coated 24-well plates at 3 × 10^5^ cells/well and maintained in Dulbecco's modified Eagle's medium (DMEM) with low glucose (Cat.#. 11885092, Thermo Fisher Scientific) supplemented with 5% fetal bovine serum (FBS) (Cat.#. F7524, Merck, Kenilworth, NJ, United States) for 3 days. Cells were washed twice with HBSS and incubated in 450 µl of DMEM with low glucose supplemented with 5% FBS with or without NaClO_4_ (200 µM). 0.8 MBq/ml ^125^I in 50 µl of HBSS was added to each well (*n* = 5), followed by incubation for 60 min (37°C, 5% CO_2_). The incubation buffer was removed and ^125^I uptake into the cells was immediately terminated by adding 1 ml of ice-cold HBSS.

The cells were washed twice with 1 ml of ice-cold HBSS and lysed with NaOH (0.5 N, 0.5 ml) for 60 min for protein and radioactivity measurements. The protein content of each cell lysate was measured using Pierce BCA Protein Assay Kit (Cat.#. 23225, Thermo Fisher Scientific) and a filter-based microplate photometer (Multiskan FC, Thermo Fisher Scientific). The radioactivity of each lysate was measured using a γ-counter (AccuFLEX γ7001, Nippon RayTech Co., Tokyo, Japan). Following decay correction of the cell lysate, the percentage of cell uptake radioactivity in each lysate was calculated as follows:

$$\begin{array}{r} (\mathrm{Uptake}\;\mathrm{Index})\;=\;\frac{100[\left(\mathrm{Radioactivity}\;\mathrm{in}\;\mathrm{cell}\;\mathrm{lysate}\;(\mathrm{cpm}))/\mathrm{protein}\;\;\mathrm{volume}\;\mathrm{in}\;\;\mathrm{cell}\;\mathrm{lysate}\;(\mathrm{mg}))\right]}{\mathrm{Radioactivity}\;\mathrm{in}\;\mathrm{added}\;\mathrm{radiotracer}\;(\mathrm{cpm})} \end{array}$$

### 2.4. Animal experiments

Animal experiments followed the Animal Research: Reporting of *in vivo* Experiments (ARRIVE) guidelines. All mice and rats were bred under appropriate care at Department of Animal Resources, Advanced Science Research Center, Okayama University. Isoflurane (Isoflurane Inhalation Solution, Pfizer Japan, Tokyo, Japan) was used for inhalation anesthesia. Carbon dioxide inhalation was used for euthanasia. The humane endpoint for discontinuation of the experiment was defined as a decrease in body weight of more than 20%/3 days, a decrease in water and food intake, or a decrease in locomotion, when the animal's distress was deemed intolerable. No corresponding signs were observed in this study. No randomization, no strategy used to minimize potential confounders, or no blinding were done in this study.

### 2.5. Teratoma formation assay

To evaluate teratoma formation capacity, 1 × 10^7^ NIS-expressing or NIS non-expressing human iPSCs suspended in 50% Matrigel (Cat.#. 354277, Corning)/50% StemFit AK02N with 10 µmol/L Y-27632 were injected subcutaneously into the back skin of 8-week-old male NSG mice (NOD.Cg-*Prkdc^scid^Il2rg^tm1Wjl^*/SzJ, Jackson Laboratory, Yokohama, Japan). Cell injection was performed after an acclimation period of at least one week. Six mice were used in this study. Six weeks later, teratomas were harvested after euthanasia by carbon dioxide inhalation, fixed in 10% formalin for 48 h, embedded in paraffin, and sectioned for tissue evaluation using hematoxylin and eosin staining.

To detect teratomas by SPECT/CT imaging, 1 × 10^7^ human iPSCs suspended in 50% Matrigel/50% StemFit AK02N with 10 µmol/L Y-27632 were injected subcutaneously into the back skin of 8-week-old male severe combined immunodeficiency (SCID) rats (F344-*II2rg/Rag2^em1lexas^*, NBRP-Rat with support in part by the National BioResource Project of the MEXT, Japan). Cell injection was performed after an acclimation period of at least one week. Two rats were used in this study. Six weeks later, ^99m^TcO_4_^−^ SPECT/CT was performed, and teratomas were harvested and evaluated using hematoxylin and eosin staining and immunostaining.

### 2.6. Evaluation of human induced pluripotent stem cell proliferation

Human iPSCs were seeded at 1 × 10^4^/cm^2^ on iMatrix-511-coated 24-well or 96-well plates and cultured in Essential 8 Flex Medium (Cat.#. A2858501, Thermo Fisher Scientific) or RPMI 1640 (Cat.#. 11875093, Thermo Fisher Scientific) supplemented with B-27 supplement (Cat.#. 17504044, Thermo Fisher Scientific) and sodium iodide (Cat.#. 194-02272, Fujifilm Wako Pure Chemical Corporation, Osaka, Japan) for 4 days. The cell area fraction relative to the plate surface area was measured using F-actin/nuclear staining to evaluate cell numbers. To measure the cell area fraction using ImageJ (18), the image was converted to 8-bit, “Threshold” was manually set to (20, 255), “Black background” was checked, “Convert to Mask” was clicked, and “Area fraction” was measured. In addition, MTT assay (Cat.#. M009, Dojindo Laboratories, Kumamoto, Japan) was performed. Absorbance was measured using FlexStation 3 (Molecular Devices, Sunnyvale, CA, United States).

### 2.7. Differentiation of human iPSCs to cardiomyocytes

Cardiac differentiation was performed according to the protocol reported by Lian et al. (19). Human iPSCs were dissociated into single cells using StemPro Accutase Cell Dissociation Reagent and seeded on 0.5 µg/cm^2^ iMatrix-511 silk-coated 12-well plates (Cat#. 3513, Corning) at 2.5 × 10^4^/cm^2^ in StemFit AK02N supplemented with 10 μmol/L Y-27632. The medium was replaced daily with a medium without Y-27632. Four days after plating, on day 0 of differentiation, cells were treated with 12 μmol/L CHIR99021 (Cat#. S2924, Selleck chemicals, Houston, TX, United States) in RPMI 1640 (Cat#. 11875093, Thermo Fisher Scientific) supplemented with B-27 supplement, minus insulin (Cat#. A1895601, Thermo Fisher Scientific). After 24 h, on day 1, the medium was changed to RPMI 1640 supplemented with B-27 supplement, minus insulin and 100 μmol/L ascorbic acid 2-glucoside (Cat#. AG121, Hayashibara, Okayama, Japan). On day 3, the medium was changed to RPMI 1640 supplemented with B-27 supplement, minus insulin and 5 μmol/L IWP2 (Cat#. S7085, Selleck chemicals). On day 5, the medium was changed to RPMI 1640 supplemented with B-27 supplement, minus insulin. From day 7, the medium was changed every 2–3 days. On day 12, the medium was changed to DMEM without glucose (Cat#. 11966025, Thermo Fisher Scientific) supplemented with 8 mmol/L L-(+)-Lactic acid (Cat#. L1750, Merck) to remove non-cardiomyocytes (20). After day 14, the cells were maintained in low-glucose DMEM supplemented with 5% FBS. Purified cardiomyocytes were cryopreserved with Bambanker hRM (GC Lymphotec, Tokyo, Japan) on day 17 for transplantation.

### 2.8. Evaluation of human induced pluripotent stem cell-derived cardiomyocyte proliferation/viability

Human iPSC-CMs were seeded at 2 × 10^4^/well on iMatrix-511-coated 96-well plates and cultured with DMEM, low glucose supplemented with 5% FBS and sodium iodide for 4 days. Cell Counting Kit-8 assay (Cat.#. CK04, Dojindo Laboratories) was used to evaluate cell numbers. Absorbance was measured using a FlexStation 3 (Molecular Devices, Sunnyvale, CA, United States). Cell area fraction was measured by ImageJ (18).

### 2.9. Transplantation of human induced pluripotent stem cell-derived cardiomyocytes

After an acclimatization period of at least one week, the inguinal skin of 8-week-old male SCID rats (F344-*II2rg/Rag2^em1lexa^*) was incised under isoflurane (Isoflurane Inhalation Solution, Pfizer Japan, Tokyo, Japan) inhalation anesthesia. Human iPSC-CMs at 1 × 10^7^ were suspended in MatriMix (511) with 10 µmol/L Y-27632 (Cat. #. 899001, Nippi, Tokyo, Japan) and injected into the inguinal subcutaneous adipose tissue. ^99m^TcO_4_^−^ SPECT/CT was performed 1, 6, and 10 weeks after cell injection.

After an acclimatization period of at least one week, 10-week-old male SCID rats were intubated and ventilated, and the left fourth intercostal space was opened under isoflurane inhalation anesthesia. A 7 mm-diameter aluminum rod cooled in liquid nitrogen for 1 min was placed once on the left ventricle to create cryoinjury, and 1 × 10^7^ human iPSC-CMs suspended in MatriMix (511) with 10 µmol/L Y-27632 (total volume 100 µl) were then injected into three locations around the injury. ^99m^TcO_4_^−^ SPECT/CT was performed 12 and 16 weeks after cell injection.

### 2.10. Detection of transplanted cells using ^99m^TcO_4_^−^ SPECT/CT

SPECT/CT scans were performed using a dedicated small-animal SPECT/CT system (NanoSPECT/CT, Mediso Medical Imaging Systems, Budapest, Hungary). General anesthesia was induced by using 5% isoflurane and maintained with 2% isoflurane during the experiment. The rats were injected 140–150 MBq of ^99m^TcO_4_^−^ via tail vein. The acquisition started at 30 min post-injection with a total scan time of 40 min. Following the SPECT imaging session, CT was conducted for 10 min to confirm the precise position of the injected sites. Fusion images of CT and SPECT were created, and the obtained images were analyzed using the public domain tool such as AMIDE imaging software (A Medical Imaging Data Examiner) (21). ^99m^TcO_4_^−^ uptake was calculated by multiplying the volume of regions of interest with increased ^99m^TcO_4_^−^ uptake (mm^3^) and its mean uptake value and normalizing with both the injected dose (MBq) and body weight (g) of the rat. The uptake of radioactive tracer was quantitatively evaluated in four rats for which SPECT/CT could be performed over time. Eight rats (inguinal injection, 1; heart injection, 7) that died before SPECT/CT imaging, 5 rats (inguinal injection, 1; heart injection, 4) that died during SPECT/CT imaging, and 1 rat (inguinal injection) that died after the first SPECT/CT imaging were excluded from the analysis.

### 2.11. Immunocytochemistry

The cells were plated on iMatrix-511-coated plates and fixed in 4% paraformaldehyde (Cat.#. 09154-85, Nacalai, Kyoto, Japan) or 100% cold methanol (Cat.#. 137-01823, Fujifilm Wako Pure Chemical Corporation) for 15 min. The cells were permeabilized with 0.1% Triton X-100 (Cat.#. T8787, Sigma-Aldrich, St. Louis, MO, United States) in PBS and blocked with 10% goat serum (Cat.#. 16210064, Thermo Fisher Scientific). Primary and secondary antibodies were diluted in 0.1% Triton X-100 in PBS containing 5% goat serum. The cells were stained with Hoechst 33,342 (Cat.#. H3570, Thermo Fisher Scientific) at 1 µg/ml, rabbit anti-OCT4 IgG (Cat.#. sc-9081, Santa Cruz Biotechnology, Dallas, TX, United States) diluted at 1:500, mouse anti-SSEA-4 IgG (MAB4304, Sigma-Aldrich) diluted at 1:200, mouse anti-TRA-1-60 IgM (MAB4360, Sigma-Aldrich) diluted at 1:200, mouse anti-TRA-1-81 IgM (MAB4381, Sigma-Aldrich) diluted at 1:200, mouse anti-cardiac troponin T monoclonal IgG1 (Cat.#. GTX28295, GeneTex, Irvine, CA, United States) diluted at 1:10,000, mouse anti-α-actinin monoclonal IgG1 (Cat.#. A7811, Sigma-Aldrich) diluted at 1:1,000, rabbit anti-MLC2v monoclonal Ig (Cat.#. ab92721, Abcam, Cambridge, United Kingdom) diluted at 1:500, rabbit anti-Connexin 43 IgG (Cat.#. 83649S, Cell Signaling Technology, Danvers, MA, United States) diluted at 1:1,000, rabbit anti-NKX2-5 monoclonal IgG (Cat.#. 8972S, Cell Signaling Technology) diluted at 1:2,500, mouse anti-NIS monoclonal IgG_1_ (Cat.#. MAB3564, Sigma Aldrich) diluted at 1:2,000, mouse anti-Ki67 IgG_1_ (Cat.#. 550609, BD Pharmingen, Franklin Lakes, NJ, United States) diluted at 1:1,000, and cardiac troponin I (Cat.#. sc-15368, Santa Cruz Biotechnology) diluted at 1:500. The cells were incubated with a secondary antibody, goat anti-mouse or rabbit polyclonal IgG conjugated with Alexa Fluor 488, 555, 568 or 647 (Thermo Fisher Scientific) diluted at 1:2,000, for 1 h at room temperature. The cells were observed with IX71 (Olympus, Tokyo, Japan) or BZ-X700 (Keyence, Osaka, Japan). Images were processed using ImageJ (22).

### 2.12. Immunohistochemistry

The tissues harvested after SPECT/CT were fixed with 10% formalin (Cat.#. 061-00416, Fujifilm Wako Pure Chemical Corporation) for 48 h, embedded in paraffin, and sectioned into 5-µm- thick slices using Microm HM400R Sliding Microtome (Midwest Lab Equipment, Jupiter, FL, United states). Sections were deparaffinized, immersed in citrate buffer at pH 6 or Tris-EDTA buffer at pH 9, and autoclaved at 105°C for 20 min for antigen retrieval. Mouse anti-cardiac troponin T monoclonal IgG_1_ (Cat.#. GTX28295, GeneTex) diluted at 1:4,000, rabbit anti-MLC2v monoclonal IgG (Cat.#. ab92721, Abcam) diluted at 1:2,000, mouse anti-NIS monoclonal IgG_1_ (Cat.#. MAB3564, Merck) diluted at 1:2,500, and rabbit anti-Ku80 monoclonal IgG (Cat.#. 2180S, Cell Signaling Technology) diluted at 1:800 were used for staining.

### 2.13. Statistical analysis

All data are expressed as means ± standard deviation (SD). Statistical analysis was performed by Student's *t*-test using SPSS Statistics 24 (IBM, Armonk, NY, United States). Statistical significance was set at *p* < 0.05.

## 3. Results

### 3.1. Generation of *SLC5A5* transgenic human iPSC lines

**Figure 1:**
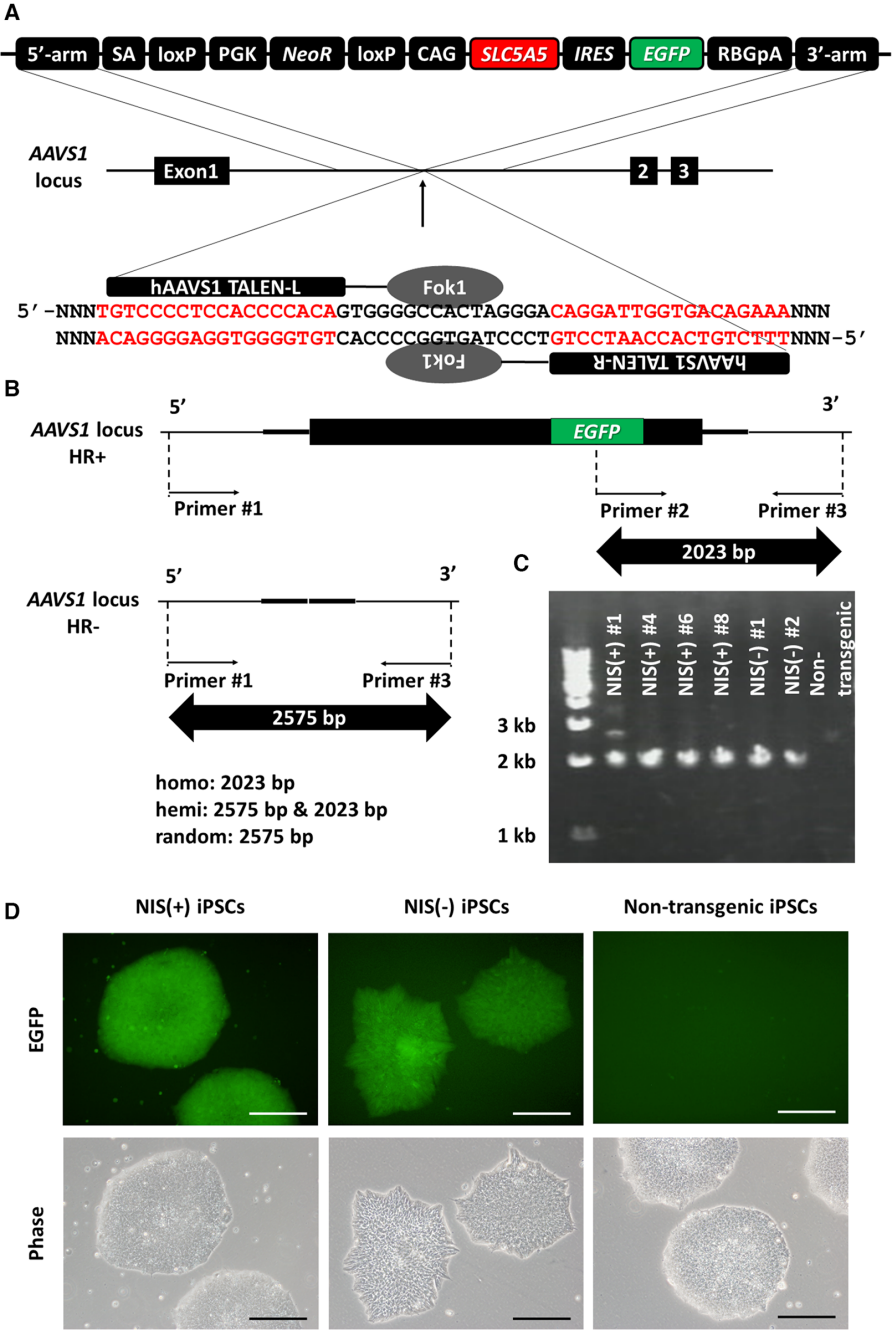
Generation of *SLC5A5* transgenic human induced pluripotent stem cell (iPSC) lines. (**A**) Transduction of human *SLC5A5* gene to *AAVS1* locus in human induced pluripotent stem cells (iPSCs) using TALEN. (**B**) The difference in size of polymerase chain reaction (PCR) product depends on the presence or absence of the transgene. (**C**) Determination of the presence or absence of gene transfer to the *AAVS1* locus using PCR of DNA extracted from each iPSC line. (**D**) EGFP expression in each human iPSC line. Scale bars are 200 µm.

To prevent transgene silencing during iPSC differentiation, the *SLC5A5* gene encoding NIS was transduced into the *AAVS1* locus known as a safe harbor (22–24) using TALEN (Figure 1A). *EGFP* and *SLC5A5* were transduced together to determine the presence or absence of NIS expression using fluorescence microscopy. Gene knock-in to the *AAVS1* locus was confirmed using PCR, and human iPSC lines with transgenes in both alleles were used in subsequent experiments: NIS-expressing human iPSC lines, NIS (+) #4, #6 and #8; NIS non-expressing human iPSC lines, NIS (−) #1 and #2 (Figures 1B,C). Fluorescence microscopy confirmed EGFP expression in the transgenic human iPSC lines (Figure 1D).

### 3.2. Expression of functional NIS in human iPSCs

**Figure 2:**
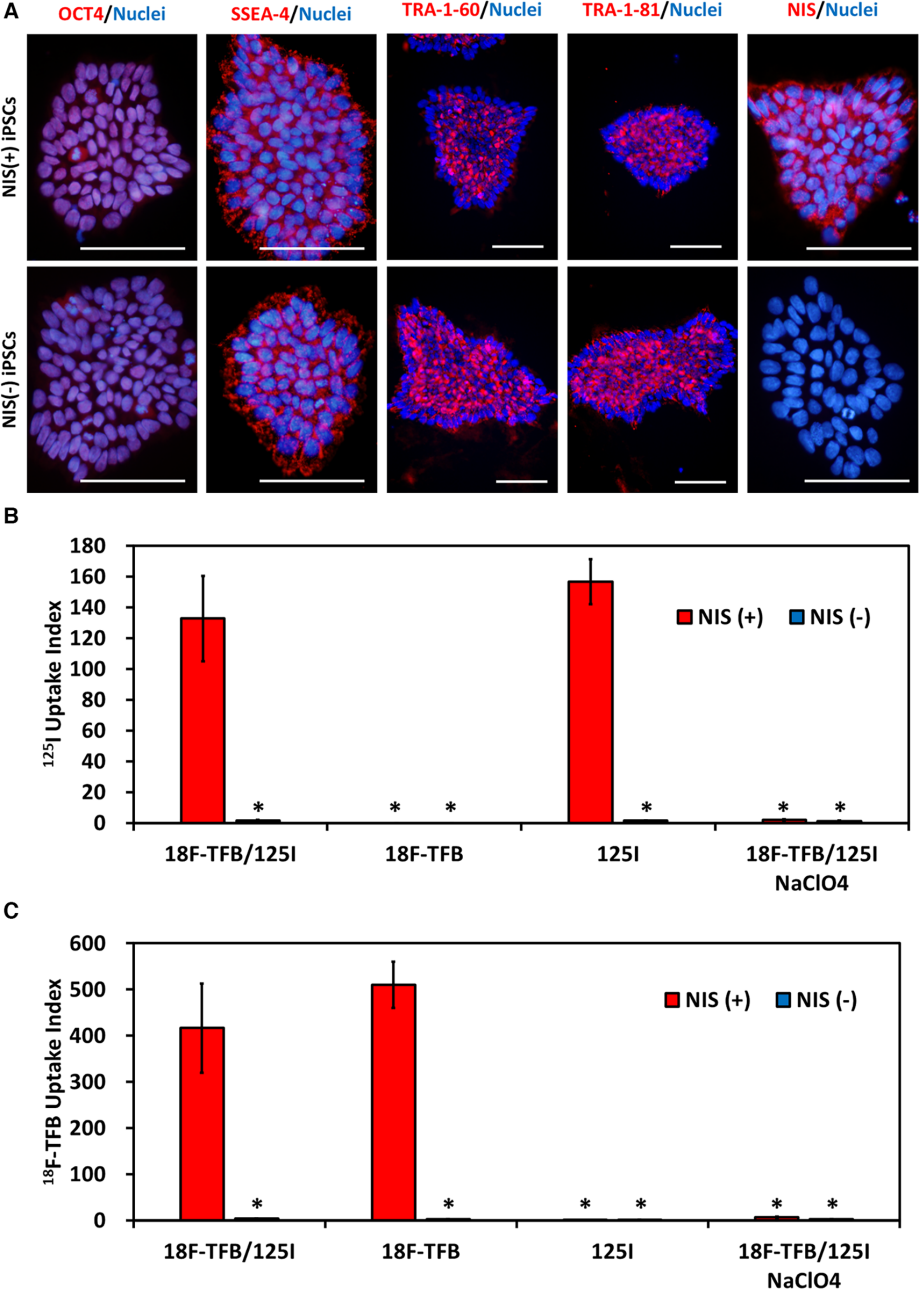
NIS-expressing human induced pluripotent stem cells (iPSCs). (**A**) Immunostaining of immature markers and NIS in human iPSCs. Scale bars are 100 µm. (**B**) Uptake of ^125^I *in vitro*. *N* = 3 in each, *p < 0.001 vs. NIS (+)/^18^F-tetrafluoroborate (TFB) and ^125^I (ANOVA/Bonferroni). (**C**) Uptake of ^18^F- TFB *in vitro*. NaClO_4_ is an inhibitor of NIS. *N* = 3 in each, *p < 0.001 vs. NIS (+)/^18^F-TFB and ^125^I (ANOVA/Bonferroni).

Undifferentiated markers of NIS-expressing human iPSCs and NIS non-expressing human iPSCs were confirmed by immunostaining. NIS protein expression was confirmed by immunostaining, and the signal was detected only in NIS-expressing human iPSCs (Figure 2A). *In vitro* radioactive tracer uptake experiments were performed to reveal the functionality of the expressed NIS. NIS-expressing human iPSCs acquired the ability to take up ^125^I and ^18^F-TFB. This uptake was completely inhibited by NaClO_4_, an inhibitor of NIS, confirming that the uptake of these radioactive tracers was due to the transduced NIS (Figures 2B,C).

### 3.3. *In vivo* detection of teratomas derived from NIS-expressing human iPSC lines by ^99m^TcO_4_^−^ SPECT/CT

**Figure 3:**
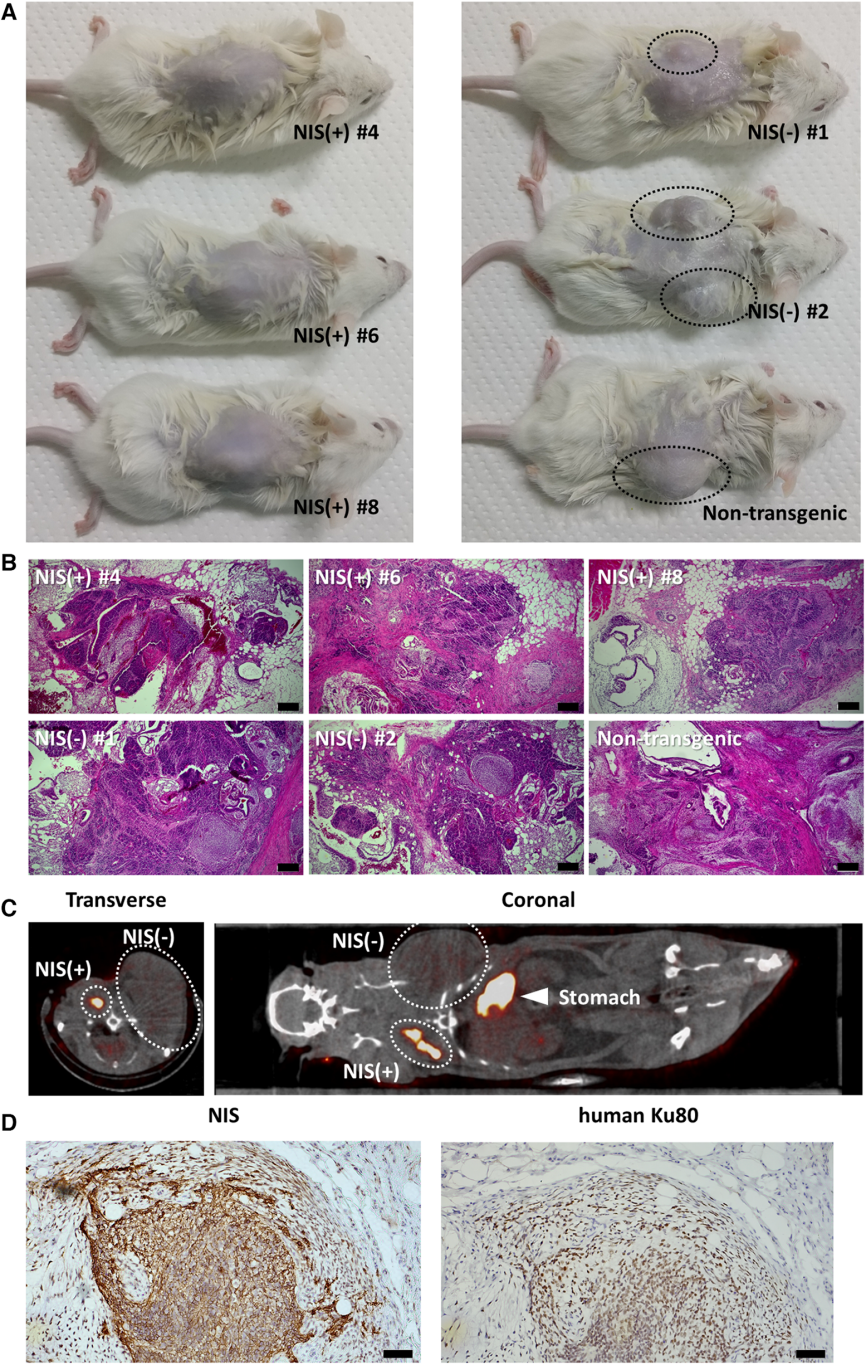
Teratoma formation assay of NIS-expressing human induced pluripotent stem cell (iPSC) lines and NIS non-expressing human iPSC lines. (**A**) Three cell lines per group were injected subcutaneously into the back in immunodeficient mice. Mice injected with NIS non-expressing human iPSC lines showed obvious mass formation after 6 weeks (surrounded by a black dashed line). In contrast, mice injected with NIS-expressing human iPSC lines showed no visible mass formation from the external surface. (**B**) Teratoma formation was confirmed histologically in NSG mice injected with NIS-expressing and NIS non-expressing human iPSC lines. Scale bars are 200 µm. (**C**) NIS-expressing human iPSCs were injected into the right side of the back and NIS non-expressing human iPSCs into the left side of the back in immunodeficient rats. Six weeks later, ^99m^TcO_4_^−^ SPECT/CT was performed to detect NIS-expressing human iPSC-derived cells. NIS-expressing human iPSC-derived cells formed a smaller mass than NIS non-expressing iPSC-derived cells (surrounded by a white dashed line). (**D**) NIS-expressing human cells were confirmed histologically in immunodeficient rats. Scale bars are 50 µm.

NIS-expressing human iPSCs and NIS non-expressing human iPSCs were transplanted subcutaneously into the back of immunodeficient mice and observed 6 weeks later. NIS non-expressing human iPSC-injected mice showed grossly visible mass formation. In contrast, mice injected with NIS-expressing human iPSCs showed no obvious mass formation on the external surface (Figure 3A). When the skin at the injection site was cut open, a small mass was observed in NIS-expressing human iPSC-injected mice, and histological analysis confirmed that it was a teratoma (Figure 3B).

Next, NIS-expressing human iPSCs were injected into the right side of the back and NIS non-expressing human iPSCs into the left side of the back of immunodeficient rats, respectively, and ^99m^TcO_4_^−^ SPECT/CT was performed 6 weeks later. As with transplantation into mice, NIS-expressing human iPSC-derived teratomas were smaller than NIS non-expressing human iPSC-derived teratomas, but uptake of radioactive tracer was observed only on the right side of the back (Figure 3C). Histological analysis confirmed that NIS-expressing human cells formed teratomas (Figure 3D).

### 3.4. Effect of NIS overexpression on cell proliferation in human iPSCs

**Figure 4:**
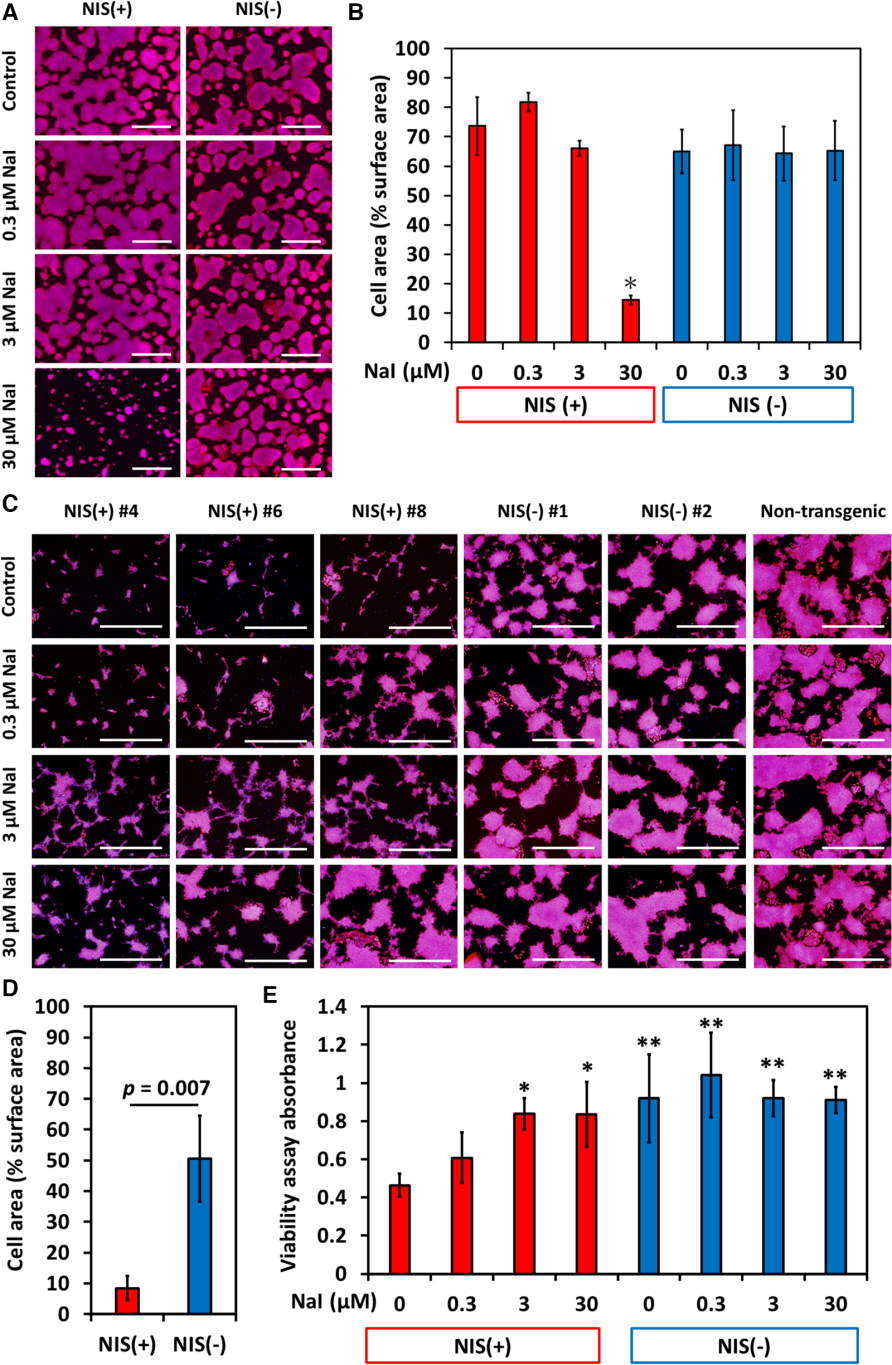
NIS-expressing human induced pluripotent stem cell (iPSC) lines showed slower proliferation in differentiation medium than NIS non-expressing human iPSC lines did. Sodium iodide (NaI) rescued poor proliferation of NIS-expressing cells. (**A**) NIS-expressing human iPSCs grew as well as NIS non-expressing human iPSCs in Essential 8 Flex Medium (maintenance medium for human pluripotent stem cells). Cells were cultured in Essential 8 Flex Medium for 4 days with or without NaI, and nuclei (blue) and F-actin (red) were stained. Scale bars are 1 mm. (**B**) Cell area/surface area was measured (*N* = 3 in each). *p < 0.001 (ANOVA/Bonferroni). (**C**) Cells were cultured with RPMI/B27 medium (basal medium for differentiation) for 4 days with or without NaI, and nuclei (blue) and F-actin (red) were stained. Scale bars are 1 mm. (**D**) Cell area/surface area was measured (*N* = 3 in each, Student-t test). (**E**) Cell counts were evaluated by MTT assay (*N* = 6 in each). *p < 0.01, **p < 0.001 vs. NIS (+) w/o NaI (ANOVA/Bonferroni).

The reason why overexpression of NIS reduces the size of teratomas was investigated *in vitro*. NIS-expressing human iPSCs grew well in Essential 8 Flex Medium, the maintenance iPSC medium. However, addition of 30 µmol/L sodium iodide (NaI), the substrate of NIS, for 4 days significantly suppressed cell proliferation (Figures 4A,B). This concentration of iodine is approximately 30 times the physiological blood concentration and thus cannot explain the phenomenon seen in teratomas. In contrast, when iPSCs were cultured for 4 days with RPMI 1640/B-27 supplement medium, a commonly used medium for differentiation, NIS-expressing iPSC-derived differentiated cells showed a marked decrease in proliferation. In addition, the addition of NaI rescued the inhibition of their cell proliferation (Figures 4C–E).

### 3.5. Characteristics of NIS-expressing human iPSC-CMs

**Figure 5:**
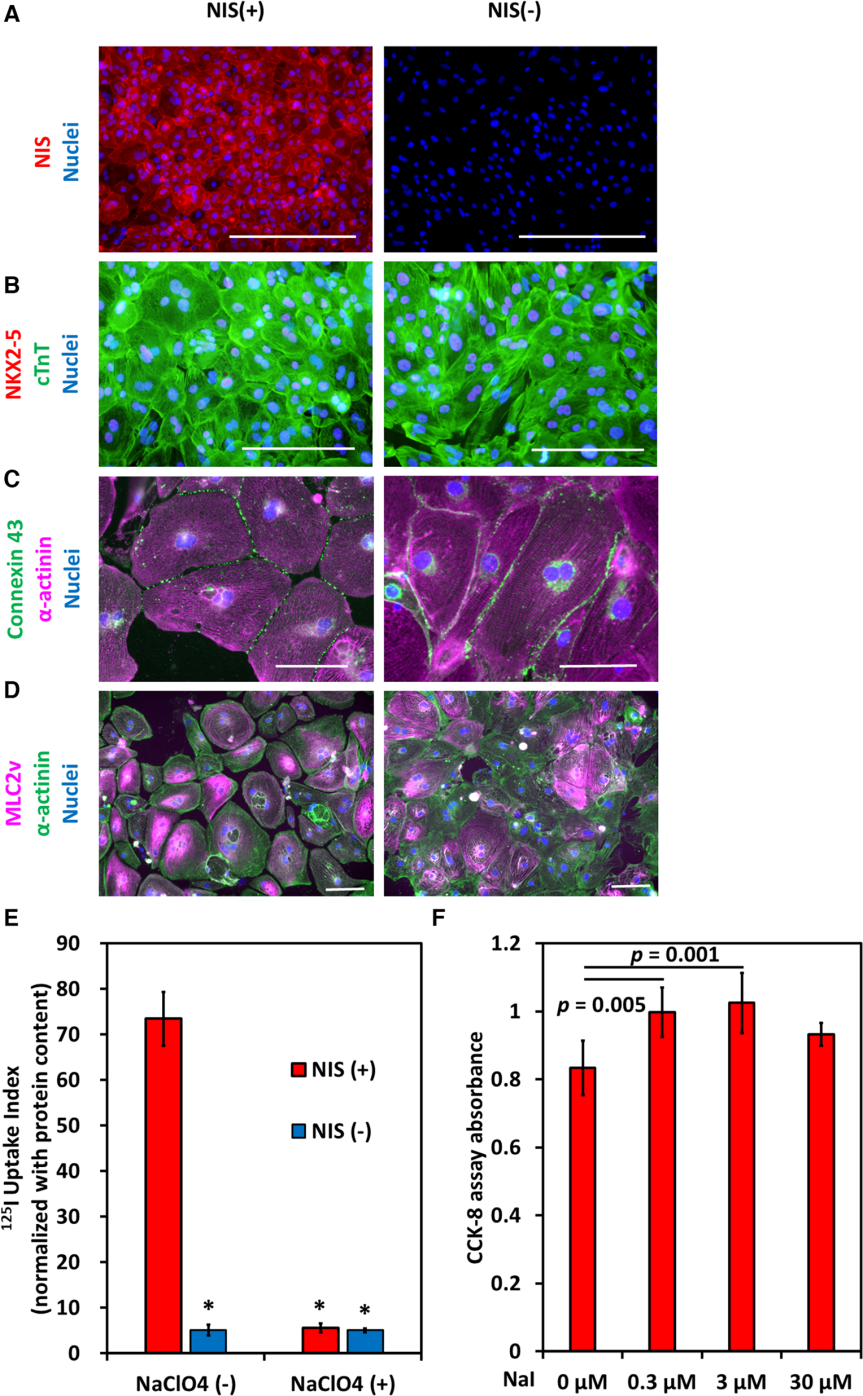
Induction of NIS-expressing human pluripotent stem cell-derived cardiomyocytes (iPSC-CMs). (**A**) Immunostaining of NIS in NIS-expressing and NIS non-expressing human iPSC-CMs at differentiation day 31. Scale bars are 400 µm. (**B**) Immunostaining of NKX2-5, a marker of working cardiomyocytes, and cardiac troponin T (cTnT) in NIS-expressing and NIS non-expressing human iPSC-CMs at differentiation day 31. Scale bars are 200 µm. (**C**) Immunostaining of α-actinin and Connexin 43 in NIS-expressing and NIS non-expressing human iPSC-CMs. Scale bars are 100 µm. (**D**) Immunostaining of α-actinin and MLC2v, a marker of ventricular cardiomyocytes, on differentiation day 50 in NIS-expressing and NIS non-expressing human iPSC-CMs. Scale bars are 100 µm. (**E**) Uptake of ^125^I of human iPSC-CMs at differentiation day 35 *in vitro*. NaClO_4_ is an inhibitor of NIS. Data are shown as mean ± standard deviation (*n* = 5 in each). *p < 0.001 vs. NIS (+) w/o 200 µM NaClO_4_ (ANOVA/Bonferroni). (**F**) NIS-expressing human iPSC-CMs were cultured for 10 days from differentiation day 21, and cell viability was evaluated using CCK-8 assay. Data are shown as mean ± standard deviation (*n* = 6 in each, ANOVA/Bonferroni).

Cardiomyocytes were induced from NIS-expressing human iPSCs and NIS non-expressing human iPSCs. After purification with no glucose DMEM supplemented with lactic acid, most cells expressed NIS (Figure 5A), working cardiomyocyte markers (NKX2-5 and Connexin 43), pan-cardiomyocyte markers (cardiac Troponin T, and *α*-actinin), and ventricular cardiomyocyte marker (MLC2v) (Figures 5B–D). NIS-expressing human iPSC-CMs were capable of ^125^I uptake, which was inhibited by the NIS inhibitor NaClO_4_ (Figure 5E). The effect of iodine on cell proliferation was examined. The addition of 0.3 and 3 µmol/L NaI for 10 days significantly increased the number of NIS-expressing human iPSC-CMs compared to NIS non-expressing human iPSC-CMs (Figure 5F). In contrast, no effect of NaI on cell number was observed in NIS non-expressing human iPSC-CMs (Supplementary Figures S1A,B).

### 3.6. *In vivo* detection of transplanted NIS-expressing human iPSC-CMs by ^99m^TcO_4_^−^ SPECT/CT

**Figure 6:**
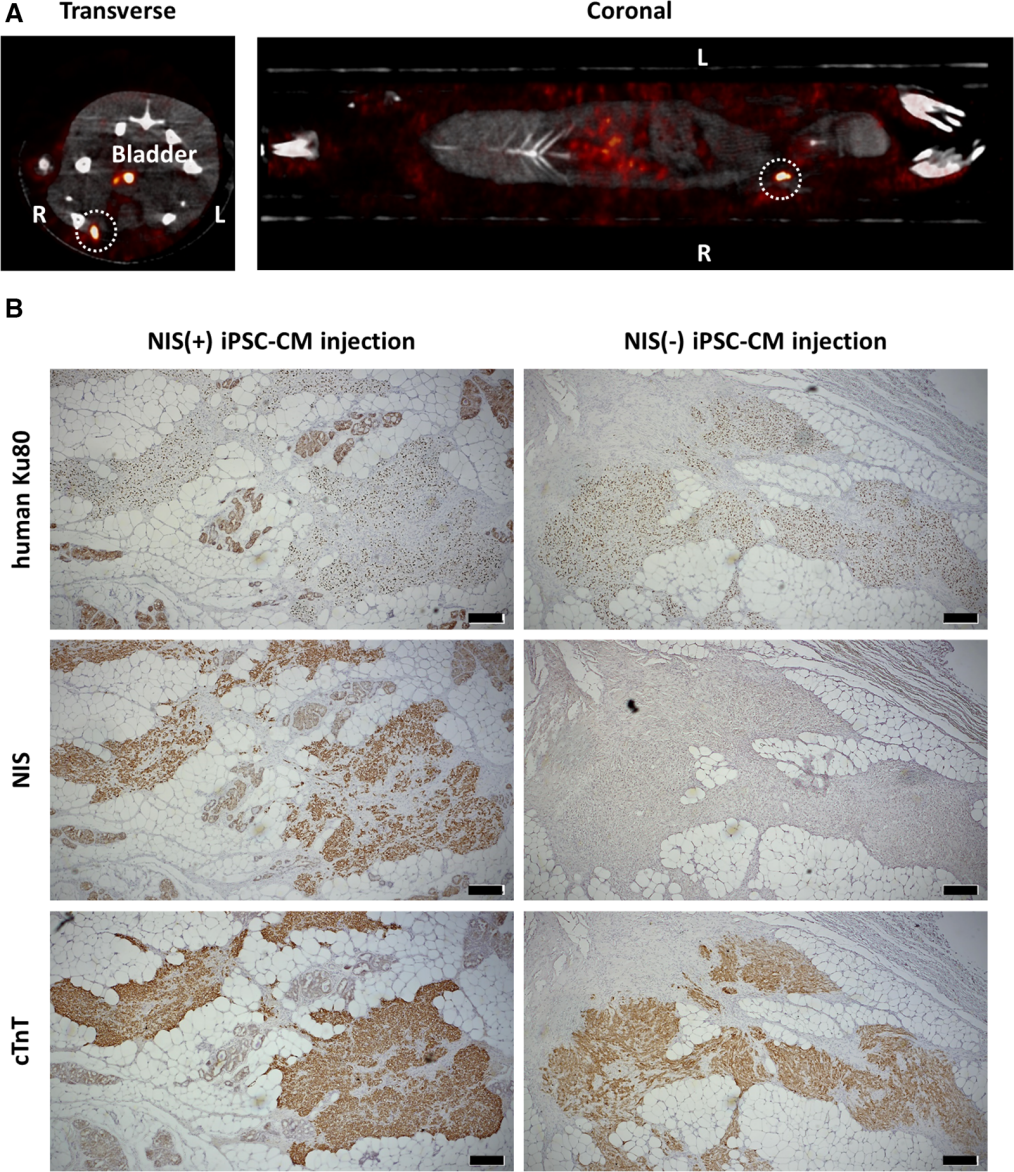
Detection of transplanted human pluripotent stem cell-derived cardiomyocytes (iPSC-CMs). (**A**) NIS-expressing human iPSC-CMs were injected into the right inguinal subcutaneous adipose tissue and NIS non-expressing human iPSC-CMs into the left inguinal subcutaneous adipose tissue in an immunodeficient rat. One week later, ^99m^TcO_4_^−^ SPECT/CT was performed to detect NIS-expressing human iPSC-CMs (surrounded by a white dashed line). (**B**) Engraftment of both NIS-expressing human iPSC-CMs and NIS non-expressing human iPSC-CMs were histologically confirmed by immunostaining of human Ku80, NIS and cardiac troponin T (cTnT). Scale bars are 200 µm.

NIS-expressing human iPSC-CMs were injected into the right inguinal subcutaneous adipose tissue and NIS non-expressing iPSC-CMs into the left inguinal subcutaneous adipose tissue of an immunodeficient rat, and ^99m^TcO_4_^−^ SPECT/CT was performed one week later. Radioactive tracer uptake was detected only in the right inguinal subcutaneous adipose tissue (Figure 6A). Histologically, human cardiomyocytes were detected in the subcutaneous adipose tissue of the bilateral inguinal regions (Figure 6B).

### 3.7. *In vivo* tracking transplanted NIS-expressing human iPSC-CMs using ^99m^TcO_4_^−^ SPECT/CT

**Figure 7:**
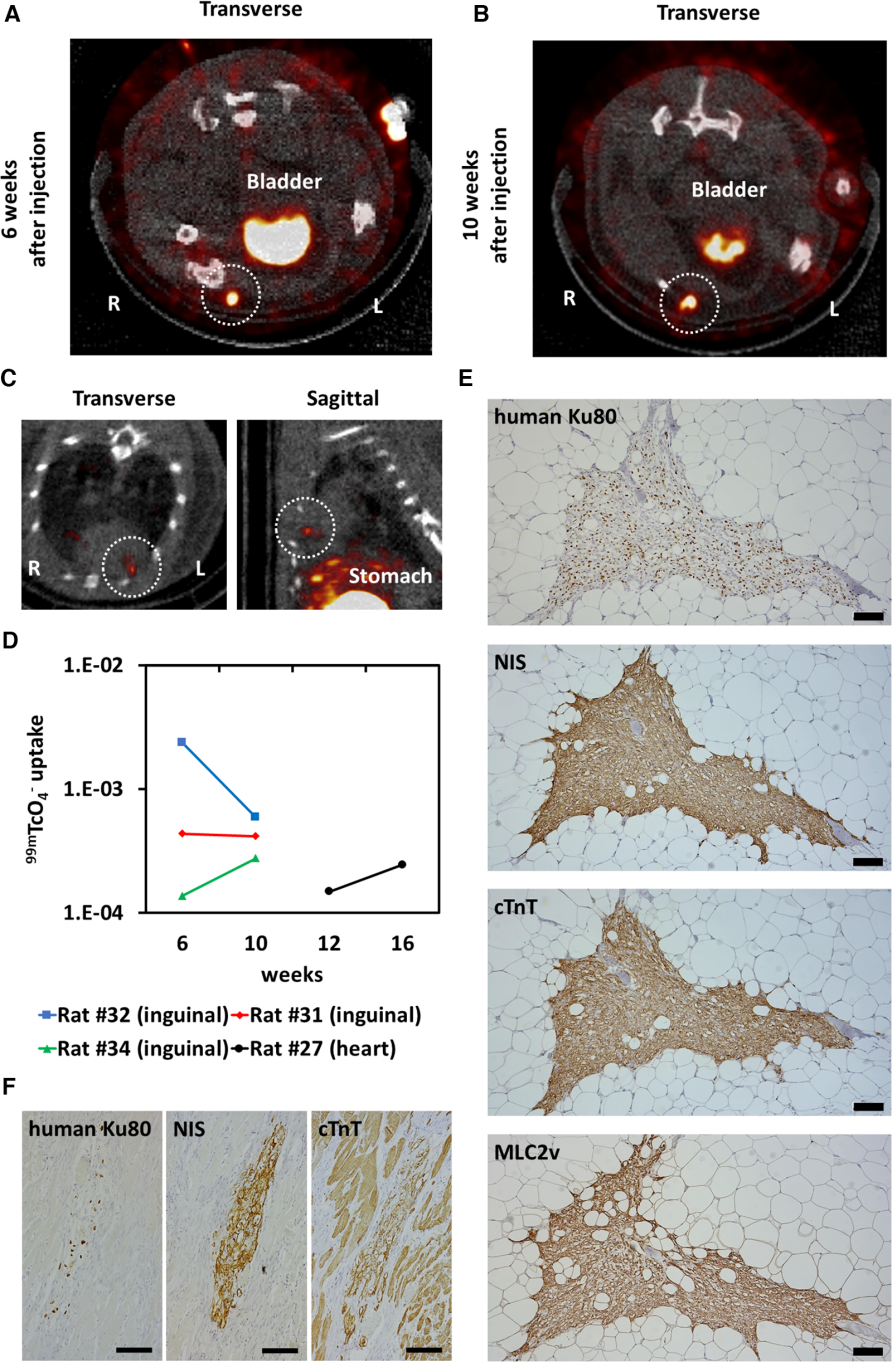
Long-term tracking transplanted human induced pluripotent stem cell-derived cardiomyocytes (iPSC-CMs). (**A**) Six weeks after injection of NIS-expressing human iPSC-CMs into the right inguinal subcutaneous adipose tissue of an immunodeficient rat, ^99m^TcO_4_^−^ SPECT/CT was performed to detect the injected cells (surrounded by a white dashed line). (**B**) ^99m^TcO_4_^−^ SPECT/CT image of the same rat shown in (**A**) 10 weeks after cell injection. Tracer uptake was observed in the right inguinal subcutaneous adipose tissue (surrounded by a white dashed line). (**C**) Sixteen weeks after injection of NIS-expressing iPSC-CMs into the cryoinjured heart of an immunodeficient rat, ^99m^TcO_4_^−^ SPECT/CT was performed to detect the injected cells (surrounded by a white dashed line). (**D**) Quantification of ^99m^Tc signals from transplanted cells over time. (**E**) Engraftment of NIS-expressing human iPSC-CMs was histologically confirmed by immunostaining of human Ku80, NIS, cardiac troponin T (cTnT), and MLC2v. Scale bars are 100 µm. (**F**) Engraftment of NIS-expressing human iPSC-CMs was histologically confirmed by immunostaining of human Ku80, NIS and cTnT. Scale bars are 100 µm.

NIS-expressing human iPSC-CMs were injected into the right inguinal subcutaneous adipose tissue of immunodeficient rats, and ^99m^TcO_4_^−^ SPECT/CT was performed 6 and 10 weeks after transplantation, showing uptake of radioactive tracer at the transplant site over time (Figures 7A,B). NIS-expressing human iPSC-CMs were injected into the heart, and ^99m^TcO_4_^−^ SPECT/CT showed uptake of radioactive tracer in the heart over time (Figure 7C). The ^99m^Tc signal was measured and the uptake value was assessed over time (Figure 7D). The same number of cardiomyocytes were administered, but the variation in ^99m^TcO_4_^−^ uptake is likely to be large. In the histological evaluation, almost all of the transplanted cells were NIS-positive and cardiac troponin T-positive. Therefore, this variation appears to be more a matter of dosing technique than a possible reduction in uptake due to decreased NIS expression (Figures 7E,F).

## 4. Discussion

This study showed that NIS-transduced human iPSCs and iPSC-derived cells acquired radioactive tracer uptake ability to track iPSC-derived cells over time using ^99m^TcO_4_^−^ SPECT/CT after transplantation into immunodeficient rats. Ostrominski et al. reported that the transduction of NIS into rhesus iPSCs can detect the engraftment of rhesus iPSC-derived cells using PET/CT after transplantation into immunodeficient mice (13). The study differed from ours by the species of iPSCs, the species of recipient animals, and the detection modalities.

This study analyzed NIS-transduced human iPSCs for future clinical applications. We demonstrated that *in vivo* imaging using NIS transduction is possible even in human iPSC-CMs. In addition, we confirmed the viability of orthotopically or ectopically transplanted human iPSC-CMs in rats, which are larger than mice. Moreover, we used ^99m^TcO_4_^−^ SPECT/CT to detect engrafted NIS-expressing human iPSC-CMs. Although SPECT/CT is less sensitive than PET/CT, it has the advantage of simultaneous multi-radionuclide imaging, which can provide information on myocardial blood flow and metabolism simultaneously (11, 25–27). In addition, it is more convenient than PET/CT due to the lower cost of radioactive tracers and its widespread use in numerous facilities. Therefore, we confirmed that ^99m^TcO_4_^−^ SPECT/CT can detect transplanted human iPSC-CMs. The ability to noninvasively detect and quantify transplanted cardiomyocytes is expected to provide the information necessary to determine the efficacy of cell therapy and to decide on treatment strategies.

The degree of tracer uptake showed no clear increasing or decreasing trend over time. This is similar to the report by Ostrominski et al. who detected NIS-expressing rhesus iPSC-CMs by PET/CT (13). In contrast, Funakoshi et al. detected luciferase-expressing human iPSC-CMs by BLI and found that the signal intensity remained enhanced from 2 weeks to 3 months after transplantation (8), suggesting proliferation of the transplanted cells. There are differences in the method of induction of cardiomyocytes from iPSCs between these studies, but it may be necessary to consider the effect of transgenes on cardiomyocyte proliferation *in vivo*.

Interestingly, the effect of NIS transduction on cell proliferation varied by the cell type. NIS-expressing human iPSCs grew as well as NIS non-expressing human iPSCs in maintenance medium, and their proliferation was not affected by physiological iodine concentrations (28). However, the addition of high concentration of NaI inhibited their growth. In contrast, the proliferation of NIS-expressing human iPSC-derived differentiated cells was inhibited when cultured in RPMI 1640/B-27 supplement medium, a commonly used basic medium for differentiation. The growth inhibition was partially rescued by adding NaI. Furthermore, human iPSC-CMs showed a slight but significant increase in cell number with the addition of NaI, corresponding to physiological iodine concentrations. Although the details of these mechanisms are unclear, it is suggested that overexpressed NIS affects the cell cycle at each differentiation stage. In addition, NIS-expressing human iPSCs formed small masses in the teratoma formation test. Histological study showed that NIS-expressing human iPSC-derived small mass contained endodermal, mesodermal and ectodermal tissues as shown in Figure 3B. This means that pluripotency is maintained in NIS-expressing human iPSCs. This result suggests the potential benefit of reducing the risk of tumor formation by undifferentiated cells intermingled with transplanted cells. Previous studies did not mention these characteristics (11, 13, 29). Templin et al. reported that no teratoma or other obvious tumor formation was detected in pig infarcted hearts injected with NIS-expressing human iPSCs (11). Moreover, Guerrieri et al. reported that endogenous and exogenous NIS promoted apoptosis in liver cancer cell lines (30). Therefore, the effects of NIS overexpression on characteristics of human iPSCs and iPSC-CMs require further investigation.

Threre are some limitations in this study. First, this study did not adequately examine human iPSC-CM transplantation into the heart. Unfortunately, most *II2rg/Rag2*-knockout rats that received cell injection into the heart died intraoperatively or during SPECT/CT imaging under inhalation anesthesia, making evaluation difficult. This may be a technical problem, but our experience with *II2rg/Rag2*-knockout rats used in this study gave us the impression that they had a higher mortality rate during and after open heart surgery than the commonly used Wistar rats (data not shown). Compared to *Il2rg*-knockout rats, *Il2rg/Rag2* knockout rats are characterized by their inability to produce natural killer cells (31). We do not know if this is related to the cause of the high intraoperative mortality rate in this study. The use of F344/NJcl.Cg-*Foxn1^rnu^* (32) and F344-*Il2rg^em7Kyo^* (33) for cell transplantation into the heart has been reported, so the use of these rats may advance research on cell transplantation study into the heart. Second, the number of cells at the detection threshold was not studied. Ostrominski et al. showed that the *in vivo* detection threshold using PET/CT was 1 × 10^5^ NIS-expressing rhesus iPSC-CMs injected into mice. Since the sensitivity of SPECT is lower than that of PET, the detection threshold is presumed to be higher than the number of cells mentioned above. Since the number of human iPSC-CMs used in clinical trials is very large (1 × 10^8^ cells) (3), it is expected that nuclear medicine imaging with NIS gene transduction will detect transplanted cardiomyocytes. Third, since the study was not performed in large animals, it is necessary to verify whether transplanted cells are detectable in large animals. However, the depth of penetration and spatial resolution of SPECT are superior to those of BLI (10, 11), and since ^99m^TcO_4_^−^ SPECT/CT is already in clinical use, clinical application of this method is expected. The pro-arrhythmic effect, which is a problem with iPSC-CM transplantation, need to be examined because NIS is electrogenic (34–37). No structural abnormalities or arrhythmias of the heart have been reported in transgenic mice with MHC promoter-driven NIS, which is specifically expressed in cardiomyocytes (38). Moreover, Ostrominski et al. showed little electrophysiological effect of transduced NIS in the range of physiological iodine concentration in monkey iPSC-CMs *in vitro* (13). However, to examine the pro-arrhythmic effect *in vivo*, transplantation experiments with large animals are necessary because rodent heart rates are too high to assess arrhythmias induced by transplanted human iPSC-CMs.

In summary, NIS-expressing human iPSC lines were established, and NIS-expressing human cardiomyocytes were induced. Transplanted NIS-expressing human iPSC-derived cells were detected using ^99m^TcO_4_^−^ SPECT/CT. ^99m^TcO_4_^−^ SPECT/CT has already been used in clinical practice, and this approach is expected to be applied clinically to confirm the viability and engraftment of transplanted human iPSC-CMs. The effects of NIS overexpression on human iPSC and iPSC-CM characteristics and functions must be studied extensively.

## Data Availability

The raw data supporting the conclusions of this article will be made available by the authors, without undue reservation.
