# Supplementary figures and images for: In vivo tracking transplanted cardiomyocytes derived from human induced pluripotent stem cells using nuclear medicine imaging
Source: Front Cardiovasc Med. 2023 Sep 7;10:1261330. doi: 10.3389/fcvm.2023.1261330 (PMC10512708; doi:10.3389/fcvm.2023.1261330)

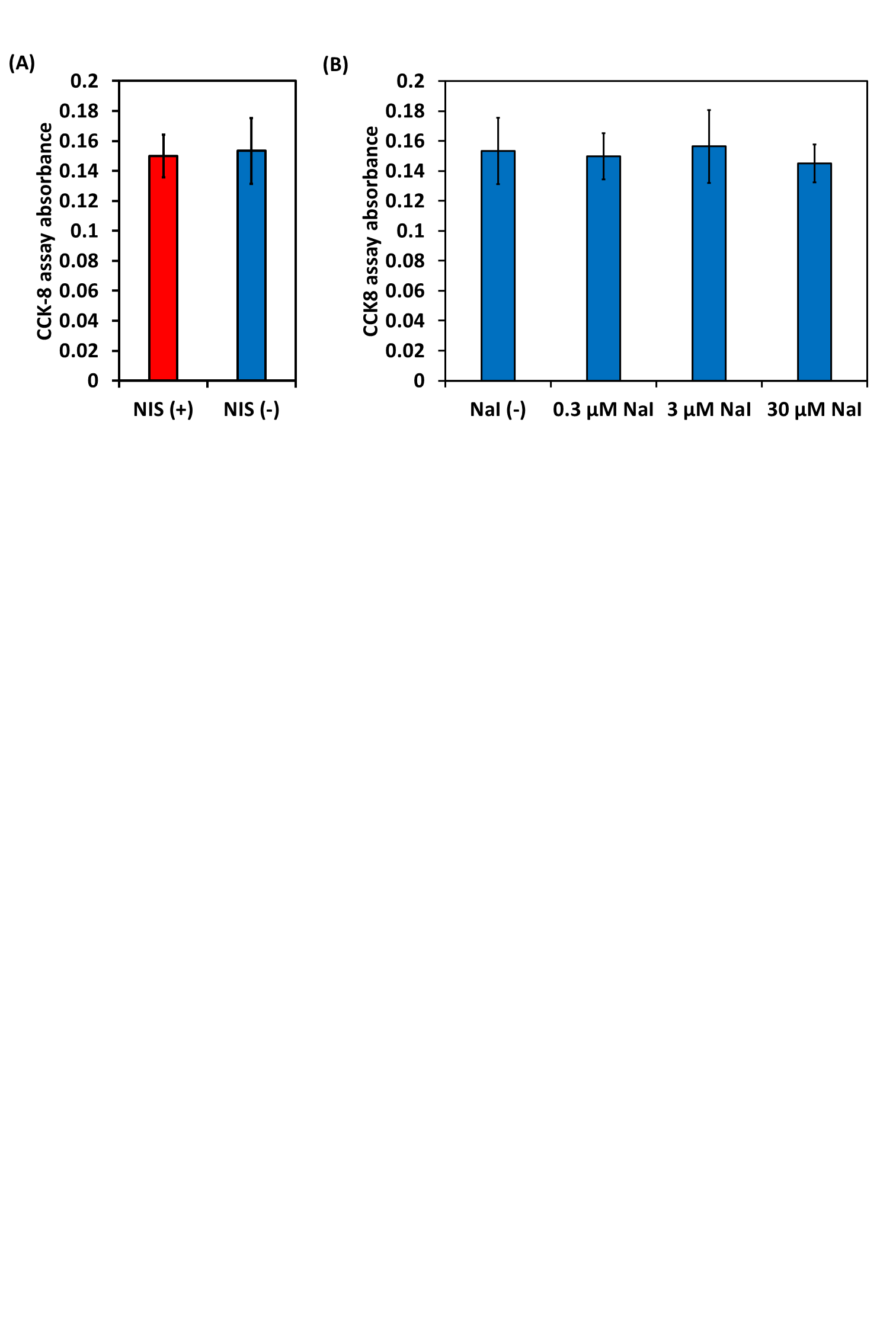

Supplement: Supplementary file 1 [file Image1.tif]
